# HPV infection and pre-term birth: a data-linkage study using Scottish Health Data

**DOI:** 10.12688/wellcomeopenres.15140.1

**Published:** 2019-03-08

**Authors:** Marian C. Aldhous, Ramya Bhatia, Roz Pollock, Dionysis Vragkos, Kate Cuschieri, Heather A. Cubie, Jane E. Norman, Sarah J. Stock

**Affiliations:** 1 Tommy’s Centre for Maternal and Fetal Health, MRC Centre for Reproductive Health, University of Edinburgh, Edinburgh, EH16 4TJ, UK; 2 HPV Research Group, Division of Pathology, Queen’s Medical Research Institute, University of Edinburgh, Edinburgh, EH16 4TJ, UK; 3 Electronic Data Research and Innovation Service (eDRIS), NHS Scotland Information Services Division, Edinburgh, EH16 4UX, UK; 4 Scottish HPV Reference Laboratory, Division of Laboratory Medicine, NHS Lothian, Royal Infirmary of Edinburgh, Edinburgh, EH16 4SA, UK; 5 Usher Institute of Population Health Sciences and Informatics, University of Edinburgh, Edinburgh, EH16 4UX, UK

**Keywords:** Pregnancy, Preterm Birth, Human Papilloma Virus, Cervix, Data linkage

## Abstract

**Background: **We aimed to investigate whether infection with high-risk (HR) types of human papilloma virus (HPV) or HPV-associated cervical disease were associated with preterm birth (<37 weeks gestation). In a sub-group of younger women who were eligible for the HPV vaccine, we aimed to determine whether prior vaccination against the specific HPV-types, HPV-16 and -18 modified preterm birth risk.

**Methods: **This was a data-linkage study, which linked HPV-associated viral and pathological information (from the Scottish HPV Archive) from women aged 16-45 years to routinely collected NHS maternity- and hospital-admission records from 1999–2015. Pregnancy outcomes from 5,598 women with term live birth (≥37 weeks gestation, n=4,942), preterm birth (<37 weeks gestation, n=386) or early miscarriage (<13 weeks gestation, n=270). Of these, data from HPV vaccine-eligible women (n=3,611, aged 16-25 years) were available, of whom 588 had been vaccinated. HPV-associated disease status was defined as: HR HPV-positive no disease, low-grade abnormalities or high-grade disease.

**Results: **High-grade HPV-associated cervical disease was associated with preterm birth (odds ratio=1.843 [95% confidence interval 1.101–3.083], p=0.020) in adjusted binary logistic regression analysis, in all women, but there were no associations with HR HPV-infection alone or with low-grade abnormalities. No associations between any HPV parameter and preterm birth were seen in vaccine-eligible women, nor was there any effect of prior vaccination.

**Conclusions:** HPV-associated high-grade cervical disease was associated with preterm birth, but there were no associations with HR HPV-infection or low-grade cervical disease. Thus HPV-infection alone (in the absence of cervical disease) does not appear to be an independent risk factor for preterm birth. For women who have undergone treatment for CIN and become pregnant, these results demonstrate the need to monitor for signs of preterm birth.

## Introduction

Human papillomavirus (HPV) is a sexually transmissible virus that infects cervical cells. Normally, the virus is cleared by the immune system, becoming undetectable within two years ^1^. Persistent HPV infection causes pre-cancerous cervical intraepithelial neoplasia (CIN); left untreated, CIN can develop into cervical cancer ^2^. Over 200 HPV types exist, of which 14 are oncogenic or high-risk (HR). Two such HR types, HPV-16 and -18, are responsible for 70–80% of invasive cervical cancers ^3^. Treatment of CIN and cervical cancer is by removal of the abnormal cervical cells ^4^. 

The cervix is important for maintenance of pregnancy, with barrier and immune mechanisms that protect the growing foetus ^5^. HPV-infection may alter cervical function, possibly increasing the risk of intrauterine infections ^6^ and subsequent complications such as preterm birth ^7^ or miscarriage ^8^.

The relationships between HPV infection and pregnancy outcomes are unclear. Some studies have shown no association ^9, 10^, whereas others have suggested that HPV-infection, cervical disease and/or its treatment are associated with adverse pregnancy outcomes, such as miscarriage ^11^, premature preterm rupture of membranes (PPROM) ^12^ or spontaneous preterm birth ^13, 14^.

We performed a data-linkage cohort study, linking women with HPV viral and pathological data with their own pregnancy records. The aims for this study were to determine whether HR HPV-infection, specific HR HPV types, or the presence of HPV-associated cervical disease were associated with preterm birth (<37 weeks gestation), early miscarriage (<13 weeks gestation) or stillbirth (pregnancy loss at ≥24 weeks gestation) and whether previous vaccination against HPV-16/18 affected these outcomes.

## Methods

### Study background

This study was a data-linkage study, linking data from women with HPV viral and pathological data contained within the Scottish HPV Archive (see below) with their own pregnancy records. After data-cleaning, the retrospective data from the women in the resulting cohort were analysed for associations between the HPV viral/pathology parameters and adverse pregnancy outcomes: preterm birth (<37 weeks gestation), early miscarriage (<13 weeks gestation) and stillbirth (≥24 weeks gestation). The study population for whom data were obtained and linked were women who had samples and data within the Scottish HPV Archive and who had had details of pregnancy outcomes.

### Eligibility criteria

Women whose HPV-infection status was known at the time of birth were included. As HPV is usually cleared within 2 years ^1^ and women are advised to delay their next scheduled cervical smear until at least 12 weeks postnatally ^4^, women who had a pregnancy up to 1 year prior to the cytology date were also included. In women who had had more than one pregnancy, the pregnancy outcome nearest the cytology date was included.

Exclusion criteria for the study were: Multiple (twin) pregnancies, as these babies are more often routinely delivered preterm; therapeutic terminations, ectopic pregnancies and trophoblastic disease were also excluded.

### Definition of terms

The definitions for pregnancy outcomes were: *Term live births* were women who had a live birth at term (≥37 weeks gestation); *All preterm births* were women with births before 37 weeks’ gestation including miscarriages of ≥13 weeks gestation; *Spontaneous preterm births* were women with births prior to 37 weeks’ of gestation, including those with PPROM and miscarriages of ≥13 weeks gestation, but excluding iatrogenic preterm births (those delivered by elective caesarean section or those which were induced); *Early Miscarriage* was any spontaneous pregnancy loss before 13 weeks’ gestation; *Stillbirth* was defined as any pregnancy loss after 24 weeks’ gestation.

### Sources of patient information used for the data-linkage

***Scottish HPV Archive.*** Scotland has a cervical screening programme with population coverage of around 70% ^15^. Until June 2016, women aged 20–60 years were screened; thereafter the age-range was 25–65 years. In September 2008, a routine, school-based HPV vaccination programme for girls aged 11–13 years was introduced, with a “catch-up” immunisation programme for girls aged 13–17 years ^16^. In 2009, the Scottish HPV Archive was established as a biobank for HPV-associated research. It is a collection of collections which includes residual cervical samples from women attending routine cervical screening and colposcopy clinics, from the year 2000 to the present. Many samples within the Archive have a patient identifier (community health index [CHI] number) and associated clinical, cytology and histology results, obtained from the Scottish Cervical Cytology Recall System (SCCRS). SCCRS is the national IT system that supports the Scottish cervical screening program and contains a woman’s entire screening record ^4^. A number of samples in the Archive are annotated with HPV status as a consequence of immunisation surveillance and specific research projects ^17, 18^, using different HPV assays depending on the nature and objectives of the research projects ( Table 1). At the time of data-linkage the Scottish HPV archive contained 31,320 records from women (aged 20–60 years). The Scottish HPV Archive has generic Research Tissue Bank approval from the East of Scotland Research Ethics Service (REC Ref 11/AL/0174) and is registered with Lothian NRS Bioresource. Approval for use of HPV data was given by the Archive Steering Committee (HPV Archive Application Ref 0018).

**Table 1.** Variable coding obtained or used for coding data. Table shows the variables obtained from the data linkage, the source of the data, the codes (ICD or otherwise) used in the original dataset and the codes used for each variable in the data analysis.

Variable name	Description and/or codes received from data sources		Source	Cleaning strategy and coding used
Unique ID	Unique identifier for mother used to link datasets			No change
Maternal age	Maternal age at pregnancy admission (years)		SMR02/ SMR01	No change
Year & month of outcome	Yyyymm		SMR02 / SMR01	No change. Used to calculate time between HPV cytology and pregnancy
Outcome of pregnancy	1=Live birth 2=Still birth 3=Livebirth dying within the first 6 days (early neonatal death) 4=Livebirth dying between 7th completed day and 28th day (late neonatal death) 5=Livebirth dying between 28th day and end of the first year of life (post-neonatal death)		SMR02 / SMR01	0=Term live birth (live birth ≥ 37 weeks) 1=Preterm birth (live birth < 37 weeks); includes late miscarriages (≥13 weeks gestation) 2=Stillbirths – **removed** 3=Neo-/post-natal death included in live birth data of appropriate gestation 4=Miscarriage. Miscarriages from SMR01 coded as <13 weeks gestation (early); miscarriages from SMR02 were coded as early (<13 weeks) or late (≥13 weeks) by gestation 5=live birth of unknown gestation – **removed**
Estimated Gestation	Estimated gestation at abortion or delivery (weeks) [not available for data from SMR01]		SMR02 only	Estimated gestation used to define pregnancy outcomes
Miscarriage Flag	Derived from ICD10 coding O01-O03,O05,O06, Type of Abortion, and Outcome of Pregnancy		SMR01 / SMR02	0=No 1=Yes
Type of abortion	1=spontaneous / incomplete abortion (miscarriage) 2=missed abortion (miscarriage) 3=trophoblastic disease 4=therapeutic abortion 5=suspected illegal abortion 6=ectopic pregnancy 8=other abortion 9=unspecified		SMR02	**Only present when no live birth** Any pregnancies with 1 or 2 classed as miscarriage. For values 3–9, records relating to that pregnancy were removed. Coded as miscarriage: 0=No 1=Yes
Mode of delivery	0=Normal, spontaneous vertex, vaginal delivery, occipito–anterior 1=Cephalic vaginal delivery, with abnormal presentation of the head at delivery, without instruments, with or without manipulation 2=Forceps, low application, without manipulation, forceps delivery NOS 3=Other forceps delivery. Forceps with manipulation, high forceps, mid forceps 4=Vacuum extraction ventouse 5=Breech delivery, spontaneous assisted or unspecified partial breech extraction 6=Breech extraction, NOS. 7=Elective (planned) Caesarean Section 8=Emergency / unspecified caesarean section 9=Other and unspecified method of delivery Additional codes provided were from SMR02 coding sheet: A=mid cavity forceps B=rotational forceps C=Ventouse no rotation specified D=Ventouse with rotation E=Other forceps delivery		SMR02	Coding collapsed to: 0=any vaginal delivery 1=any forceps delivery 2=any ventouse delivery 3=any breech delivery 7=Elective caesarean section (ELCS) 8=Emergency caesarean section (EmCS) 9=Missing Coding used to determine whether delivery was by elective caesarean section
Induction of labour	0=none 1=Artificial rupture of membranes (ARM) 2=Oxytocics 3=ARM + Oxytocics 4=Prostaglandins (includes cervical priming) 5=ARM + Prostaglandins 6=Prostaglandins + Oxytocics 7=Prostaglandins + ARM + Oxytocics 8=Other 9=Not Known		SMR02	0=None 1=ARM only 2=Any drugs ± ARM 9=Missing Coding used to define whether labour onset was spontaneous.
Prelabour rupture of membrane (PROM)	Derived from ICD10 code O42.		SMR02	0=No 1=Yes 99=Missing Coding used for spontaneous labour onset in preterm birth
Ethnicity	A - White (1A=Scottish, 1B=Other British, 1C=Irish, 1K=Gypsy, 1L=Polish, 1Z=other) B- Any mixed or multiple ethnic groups C - Asian (Pakistani / Indian / Bangladeshi / Chinese / Other) D - African E - Caribbean or Black F - Other Ethnic group G - Refused 98= Not given by patient H - 99 = unknown		SMR02 / SMR01	Recorded for approximately 70% of cases only Collapsed to Ethnicity code: 1=White 2=Other 99=Missing
Scottish Index of Multiple Deprivation (SIMD)	SIMD (National quintiles and deciles) SIMD provided from 2004, 2006, 2009, 2012 for each admission. SIMD scores have changed calculation methods and coding direction over time		SMR02 / SMR01	SIMD quintile for 2009 (midpoint between 2000 and 2015) was used for all records. Quintiles were combined as: 1 and 2=most deprived 3, 4 and 5 =least deprived 99=missing
Parity	0–7=number of actual events 8=8 or more actual events 9=not known Not available for SMR01. Some information if patient had another pregnancy		SMR02	0=non-parous 1=parous 99= missing
Smoking history at booking	0=never smoked 1=current smoker 2=former smoker 9=unknown/missing		SMR02	No change
Current Smoking during pregnancy	0=No 1=Yes 9=Unknown/missing		SMR02	No change
Diabetes	1=yes pre-existing (diagnosed before pregnancy) 2=yes gestational (diagnosed during pregnancy) 3= yes time of diagnosis unknown 4=no diabetes during pregnancy 9=not known		SMR02	0=No diabetes 1=any diabetes during pregnancy 9=Missing
Hypertensive disorder in pregnancy	Did not distinguish between pre-existing and gestational hypertension 0=No 1=Yes 99=unknown		SMR02	0=No 1=Yes 99=Missing
Date of Sample Collection	Date of collection Same as cytology date		HPV Archive	Year only Coded as January of year if no cytology date
Cytology date	Year and month		HPV Archive	Cytology date used to define date of HPV diagnosis Used to calculate time between cytology and pregnancy outcome
Years between cytology and pregnancy	Derived parameter		--	Calculated time between cytology and pregnancy (years). Includes cytology up to a year after pregnancy
HR HPV type	HPV types were detected. For some samples, multiple strains were detected. All positive results denote high-risk (HR) type. HR types: 16, 18, 31, 33, 35, 39, 45, 51, 52, 56, 58, 59, 66, 68. Some results given as "other HR HPV". Low risk (LR) types were only detected in samples that were specifically genotyped and classed as HR HPV negative. LR types: 6, 11, 40, 42, 43, 44, 53, 54, 61, 72, 73, 81. HR HPV assays used were Hybrid Capture 2 (Qiagen, Germany), Optiplex HR HPV test (Diamex, Germany), RealTime HR HPV assay (Abbott Molecular, USA), in house PCR assay ^21^ or Linear Array HPV genotyping test (Roche, USA). Assays for individual genotypes used Optiplex HR HPV test, RealTime HR HPV assay or Linear Array HPV genotyping test.		HPV Archive	Coded as : 0=HR HPV negative 1=HPV HR positive 9=HPV unknown If no genotype given but was positive, sample coded as HR HPV. If only LR genotypes present, sample was coded HR HPV negative For specific analysis of HPV 16/18 types, only those samples were used which had a known specific HPV type, coded as: 0=HR HPV negative 1= HPV 16/18 +ve: samples contained either or both of these types, regardless of other types contained therein. 2= non-16/non-18 HR types: samples contained known HR HPV types but not HPV-16 nor HPV-18
Cytology result	Old Codes: (before 2012) • Normal • Borderline squamous changes, mild dyskaryosis • Moderate dyskaryosis, severe dyskaryosis • Unknown	Equivalent codes (after 2012) • Normal • Low-grade dyskaryosis • High-grade dyskaryosis • Unknown	HPV Archive	Data arrived as mix of new and old codes. New codes used, corresponding to old codes as shown Used to define HPV disease
Histology date	Year and month		HPV Archive	No change
Histology result	**Samples for histology only taken if abnormal cytology** Normal, Cervical intraepithelial neoplasia (CIN) CIN1-3, Cervical glandular intraepithelial neoplasia (CGIN), high-grade dysplasia, high/low-grade intraepithelial lesion, human papilloma virus, squamous cell cancer (SCC), unsatisfactory Available for ~5% of women		HPV Archive	Coded as: 0=Normal, mild changes (HPV), unsatisfactory 1=CIN1, low-grade intraepithelial lesion, changes consistent with HPV 2=CIN2, CIN3, high-grade intraepithelial lesion, SCC, CGIN Used to define HPV disease
HPV-associated cervical disease	Derived parameter based on all HPV data and used as an HPV parameter in analysis.		--	0=HPV Neg/LR 1=HPV HR type, normal cytology 2=Low-grade disease (abnormal cytology; normal/low grade histology, CIN1) 3=High-grade disease (High grade dysplasia, CIN2, CIN3, CGIN, SCC, high-grade intraepithelial lesion) 8=HPV HR type, cytology unknown } marked as 9=HPV disease unknown } missing
HPV vaccination status	Vaccine-eligible women were those who were aged 12 from 2008 onwards, or girls up to 18 years of age who were eligible for the catch-up vaccination programme.		HPV Archive	0=Vaccine-eligible women who were not vaccinated 1=Vaccine-eligible women who were vaccinated 2= Women too old to be eligible for vaccine } marked as missing 9= Eligible women unknown if vaccinated } in VE analyses

***Scottish Morbidity Record (SMR)02.*** SMR02 is the Maternity Inpatient and Day Case Dataset ^19^, held by the Information Services Division (ISD) of the National Health Service (NHS) National Services Scotland (NSS) and covers maternity admissions in Scotland. SMR02 includes over 98% of deliveries, as less than 2% of deliveries occur at home. A quality assurance audit of SMR02 showed that accuracy of the records exceeded 90%, when compared with corresponding written medical records ^20^. 

***SMR01.*** SMR01 is the General/Acute Inpatient and Day Case Dataset, held by NHS NSS ISD, and covers hospital admissions in Scotland ^19^. Audit of SMR01 found that accuracy of clinical data was more than 89%, when compared with written medical records ^22^. Miscarriage data were obtained from both SMR01 and SMR02. If an SMR01 record had no corresponding SMR02 record, we assumed that the miscarriage occurred before 13 weeks’ gestation, as it is usual practice for pregnancies to be registered on SMR02 before 13 weeks gestation ^23^. Scottish birth statistics from the Scottish population, obtained from SMR02 and SMR01, are available from the NHS NSS ISD website, Births in Scottish Hospitals ^23^.

Miscarriage may occur before the mother is aware she is pregnant, and is often managed in an outpatient or general practice setting ^23, 24^; such cases would not generate a hospital or maternity record. Women admitted to a hospital ward via an emergency or gynaecology department or early pregnancy unit generate an SMR01 record; women admitted to a maternity unit generate an SMR02 record. Between 1998 and 2016, the population rate of miscarriages managed in Scottish hospitals (data from SMR01 and SMR02) fell from 7.0 to 4.4 per 1000 women aged 15–44 per year ^23^. From these numbers, the percentage of miscarriages in the Scottish birth data for each year was calculated as:

$$\text{Percentage}\;\text{of}\;\text{miscarriages}\;\text{=}\;\frac{\text{Number}\;\text{of}\;\text{miscarriages}\;\text{given}\;\text{for}\;\text{each}\;\text{year}}{\text{Number}\;\text{of}\;\text{miscarriages}\;\text{+}\;\text{number}\;\text{of}\;\text{births}\;\text{for}\;\text{each}\;\text{year}}$$

From this calculation, for the years 1998–2016 the percentage of miscarriages approximated to 8.0- 12.0% of all births per year.

***National Records of Scotland (NRS).*** This resource hold the records of stillbirths and infant deaths in Scotland ^25^.

### Approval for use of data

Approval for use of NHS NSS and NRS data was obtained from the NHS Privacy Advisory Committee (PAC 74/14). Approval for linkage of previous unlinked datasets was given by the NHS Scotland National Caldicott Guardian Forum (Ref 2015-19). The linked dataset was analysed within the NHS National Safe Haven, provided by NHS Research Scotland ^26^. The Safe Haven is a remote server through which the researcher accesses the health data and services to enable research while protecting the confidentiality of the data. Data remains under the control of the NHS and complies with legislative and NHS policies. The linked dataset is archived within the Safe Haven and is available by application to NHS Scotland via the electronic Data Research and Innovation Service (eDRIS) ^27^, within the Farr Institute for Health Informatics Research.

### Data extraction

**Figure 1.**
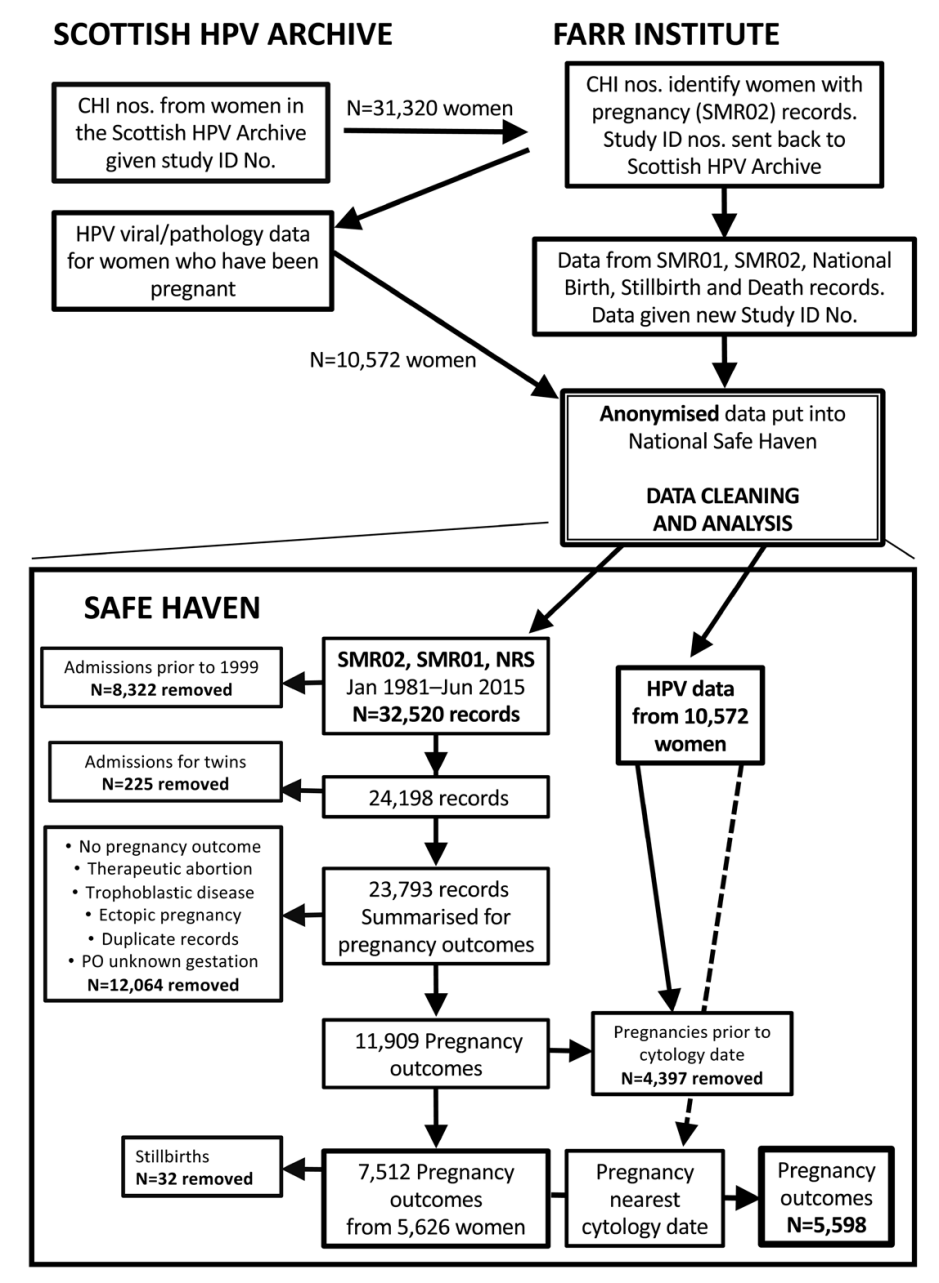
Summary of data-linkage and -cleaning processes.

The data-linkage and -cleaning processes are summarised in Figure 1. Data were transferred between the Scottish HPV Archive and eDRIS, where data-linkage was performed. From all the women’s records within the Scottish HPV Archive (N=31,320), those who had had pregnancies recorded in SMR02, SMR01 and NRS were identified by the CHI number. Coded identification numbers of women with pregnancy records were sent back to the Scottish HPV Archive (n=10,572), from where HPV-genotype, cytology date and result, histology date and result, vaccination date and dosage were collated. These data are denoted as ‘HPV viral/pathology data’. The HPV viral/pathology data and corresponding SMR02, SMR01 and NRS records were collated, anonymised and placed in the Safe Haven. For data cleaning and analysis, only the Safe Haven and the linked datasets were accessed.

Pregnancy admission records from SMR02, SMR01 and NRS records for women in the Scottish HPV Archive were received from January 1981 to August 2015. Many of the older women within the Scottish HPV Archive had their pregnancies before their HPV-infection status was known and so were excluded. Records in the Scottish HPV Archive started in 2000, so pregnancy admissions before 1999 were removed; therefore, the time-period for this study was 1999–2015. Duplicate records were removed. Information from multiple admissions per pregnancy outcome were condensed into one record. The variables obtained from SMR01, SMR02, NRS records and the Scottish HPV Archive are presented in Table 1, together with details of data coding and cleaning. Pregnancy records were coded without reference to HPV-infection status. The proportions of women with specific pregnancy outcomes within our final cohort was compared with that of the Scottish population ^23^ to determine how representative our cohort was of the Scottish population.

### Classification of individuals within the dataset

HPV-infection status of each woman was defined as *HR HPV-positive* if there was a positive result for at least one HR HPV type ( Table 1). Infections with a mixture of HPV types were common. Low-risk HPV-positive samples ( Table 1) and HPV-negative results were considered *HR HPV negative*.

Women were classed as *HPV-16/18-positive* if they had samples containing HPV-16 and/or HPV-18, irrespective of other types present. Women were classed as *non-16/non-18 HR HPV-positive* if they had samples that were negative for HPV-16 or HPV-18 but contained other known HR HPV types.

*HPV-associated cervical disease* was classed as: HR HPV-negative; HR HPV-positive no disease (i.e. normal cytology); low-grade abnormalities (borderline/mild dysplasia, normal/low-grade histology, CIN1) or high-grade disease (high-grade dysplasia, CIN2, CIN3, CGIN, cancer, high-grade intraepithelial lesion).

***HPV vaccination status.*** Some women had received the bivalent HPV vaccine ^17^ (1–3 doses) against HPV-16 and -18. Vaccine-eligible women were classified as: those who had been vaccinated, those eligible (by age) but unvaccinated, or those eligible but whose vaccination status was unavailable; women born before 1990 were too old to have been eligible for vaccination.

Scottish National guidelines require over 90% of women with HPV-associated high-grade cervical disease to have undergone excisional treatment ^4^, but data for the specific treatments used were not available.

### Determining cohort reliability

To determine how representative our cohort was of the Scottish population, the proportions of women with specific HPV viral/pathology parameters within our final cohort, were compared with a recent HPV prevalence study in women attending screening in Scotland ^28^, as HPV results are not recorded as part of routine cervical screening. Pathology data (low- and high-grade disease) were compared with data from ISD ^29^.

Potential confounders for pregnancy outcomes were obtained from SMR02 and SMR01: ethnicity, parity, Scottish Index of Multiple Deprivation (SIMD), smoking history and during current pregnancy, diabetes (pre-existing and gestational) and hypertension (any). Coding and definitions for these variables are shown in Table 1. Continuous variables obtained were maternal age and maternal BMI. Neither variable was normally distributed, nor was any transformation able to normalise the data. Maternal age (range 16–45 years) was severely skewed, with 62.9% of women in our cohort aged <25 years. As we wished to investigate the effects of vaccination, and the vaccine-eligible women were aged <25 years, we chose 20 years as the age cut-off point for the analyses, comparing those aged <20 years with those >20 years. Maternal BMI data were categorised: underweight (BMI <19.9), normal (BMI 20.0–24.9), overweight (BMI 25.0–29.9) and obese (BMI ≥30.0).

### Statistical analysis

All statistical analyses were carried out using SPSS v22 (© IBM Corporation, USA). Missing data analysis ( Table 2) of the cleaned full dataset was carried out using the ‘Multiple Imputation’ function, which runs regression models of existing data to replace missing data: i.e. the programme looks at patterns of the data available and makes probability judgements (imputations) to give estimates of the missing data. Data was missing for Scottish Index of Material Deprivation (SIMD), ethnicity, parity, hypertensive disorder, current smoking, smoking history, diabetes and maternal BMI ( Table 3). Variables were imputed together so that any potential interactions could be taken into account ^30^. For the logistic regression analyses, pooled results from five imputations are presented in the results. The HPV viral/pathology parameters (HR HPV, HPV-16/18 genotype, HPV-associated cervical disease and HPV vaccination status) were not imputed, as these were the primary predictive factors of interest for the analyses.

**Table 2.** Missing data analysis. Table shows the results of the missing data analysis (number of valid variables, number and percent missing variables) for all variables with missing data.

Variables with missing data	Valid		Missing		Total N
	N	Percent	N	Percent	
No. cases with complete data	3,123	55.8%	2,475	44.2%	5,598
No. individual data values	39,590	88.4%	5,194	11.6%	44,784

The proportions of maternal characteristics according to pregnancy outcomes or according to HPV viral/pathology parameters were compared by chi squared or Fisher’s exact test analysis and are shown in Table 4 and Table 5, respectively. Binary logistic regression analysis was used for each pregnancy outcome, with women who had had term live births as the comparator group and each HPV viral/pathology parameter as the predictive factor under analysis. Odds ratios (OR) and 95% confidence intervals (CIs) are presented from unadjusted and adjusted models in all women. To investigate the effects of prior HPV vaccination, the models were run in vaccine-eligible women, with and without vaccination status included. Adjusted models used the imputed dataset and adjusted for maternal factors that might affect pregnancy outcomes: ethnicity, SIMD, parity, age, BMI, diabetes, hypertension and current smoking.

**Table 3.** Imputation models used. Table shows the models used for imputation of the missing data. Data that were not imputed were Pregnancy outcomes as these were the primary outcomes; HPV viral/pathological data because these were the primary factors of interest; maternal age was complete.

Parameters for which missing data was imputed	Valid N	Missing		Imputed values	Model	
		N	Percent		Type	Effects
SIMD	5,562	36	0.6%	180	Logistic Regression	Parity, Hypertensive disorder, Current smoking, Smoking History, Diabetes, Ethnicity, Maternal BMI
Parity	5,504	94	1.7%	470	Logistic Regression	SIMD, Hypertensive disorder, Current smoking, Smoking History, Diabetes, Ethnicity, Maternal BMI
Maternal hypertension	5,484	114	2.0%	570	Logistic Regression	SIMD, Parity, Current smoking, Smoking History, Diabetes, Ethnicity, Maternal BMI
Current Smoking	5,226	372	6.6%	1,860	Logistic Regression	SIMD, Parity, Hypertensive disorder, Diabetes, Ethnicity, Maternal BMI
Smoking history	5,061	537	9.6%	2,685	Logistic Regression	SIMD, Parity, Hypertensive disorder, Current smoking, Diabetes, Ethnicity, Maternal BMI
Maternal diabetes	4,763	835	14.9%	4,175	Logistic Regression	SIMD, Parity, Hypertensive disorder, Current smoking, Smoking History, Ethnicity, Maternal BMI
Maternal BMI at booking (kg/m ^2^)	4,148	1,450	25.9%	7,250	Linear Regression	SIMD, Parity, Hypertensive disorder, Current smoking, Smoking History, Diabetes, Ethnicity
Ethnicity	3,842	1,756	31.3%	8,780	Logistic Regression	SIMD, Parity, Hypertensive disorder, Current smoking, Smoking History, Diabetes, Maternal BMI

**Table 4.** Maternal characteristics (original/imputed data) by pregnancy outcome: Term live birth and Any preterm birth. Table shows the numbers and percentages of women in each pregnancy outcome group compared by maternal factors that might affect pregnancy outcome. The numbers shown are from original data or the imputed data (in italics). Term live births (≥37 weeks gestation) were compared with any preterm birth (PTB, <37 weeks gestation). The original (unimputed) data is presented alongside the pooled imputed data ( *italics*) to show that the proportions of each parameter are similar between the original and imputed datasets, and the imputation process did not adversely skew the data. P values denote results from chi squared analysis of the pooled imputed data for each parameter between the two groups. Significant p values are highlighted (bold); ns denotes not significant.

Maternal Factor	Original data *		Pooled imputed data ^†^		
	Term live births, n (%)	Any PTB, n (%)	Term live births (N=4,942), n (%)	Any PTB (N=386), n (%)	P value
**Age (years) ^‡^**					
Mean age (range)	25 (16–45)	24.6 (17–43)	25 (16–45)	24.6 (17–43)	
≤20 years	1082 (21.9)	80 (20.7)	1082 (21.9)	80 (20.7)	*0.654*
>20 years	3860 (78.1)	306 (79.3)	3860 (78.1)	386 (79.3)	
**Ethnicity**					
White	3283 (98.1)	267 (97.4)	*4838 (97.9)*	*377 (97.7)*	*0.713*
Other	64 (1.9)	7 (2.6)	*104 (2.1)*	*9 (2.3)*	
**Parity**					
Non-parous	3239 (65.9)	259 (67.4)	*3256 (65.8)*	*261 (67.6)*	*0.504*
Parous	1679 (34.1)	125 (32.6)	*1686 (34.1)*	*125 (32.3)*	
**SIMD ^‖^**					
Most deprived	2464 (50.1)	208 (54.3)	*2480 (50.2)*	*211 (54.7)*	*0.091*
Least deprived	2446 (49.9)	175 (45.7)	*2462 (49.8)*	*175 (45.3)*	
**BMI ^¶^ (kg/m ^2^)**					
≤19.9 (underweight)	425 (11.0)	47 (17.2)	*593 (11.9)*	*61 (15.8)*	*0.146*
20.0–24.9 (healthy)	1639 (42.5)	115 (42.1)	*1981 (40.1)*	*152 (39.4)*	
25.0–29.9 (overweight)	1043 (27.1)	57 (20.9)	*1387 (28.1)*	*97 (25.1)*	
≥30 (obese)	745 (19.3)	54 (19.8)	*981 (19.9)*	*76 (19.6)*	
**Current Smoking**					
No	3503 (74.1)	235 (64.7)	*3642 (73.7)*	*252 (65.3)*	***<0.001***
Yes	1226 (25.9)	128 (35.3)	*1300 (26.3)*	*134 (34.7)*	
**Diabetes ^#^**					
No	4282 (98.5)	312 (95.7)	*4863 (98.4)*	*370 (95.9)*	
Any	64 (1.5)	14 (4.3)	*79 (1.6)*	*16 (4.1)*	***0.002***
Hypertension **					
No	4563 (92.3)	346 (89.6)	*4562 (92.3)*	*346 (89.6)*	*0.063*
Yes	379 (7.7)	40 (10.4)	*380 (7.7)*	*40 (10.4)*	

*For the original data, due to missing values, numbers and percentages for each maternal parameter relate to the numbers in each analysis and do not add up to the total number of each pregnancy outcome.

^†^Imputed data are the pooled results of five imputations. The numbers and percentages presented are representative of the population as a whole and represent the estimated values that would have been obtained had the dataset been complete. Imputed data were used in the logistic regression models. ^‡^Maternal age was complete. Age range of women in each group is shown. ^‖^Scottish Index of Material Deprivation (SIMD) quintiles were combined as most deprived (quintiles 1 &2) vs. least deprived (quintiles 3-5). ^¶^Body mass index (BMI) was calculated as weight (kg) divided by the square of the patient’s height (m ^2^) ^#^Diabetes includes gestational diabetes and pre-existing diabetes ( Table 1). **Hypertension did not distinguish between pre-existing or gestational hypertension within SMR02 ( Table 1)

## Results

### Cohort screening, selection and characteristics

Pregnancy admission records (N=32,520) were received for women who had been pregnant and had HPV viral/pathology data in the Scottish HPV Archive (N=10,572). After data-cleaning and exclusions, we had obtained 7,512 pregnancy outcomes from 5,626 women ( Figure 1). Of these, 32 were stillbirths, which, although these had been pre-defined as one of the pregnancy outcomes for investigation, were too few for any meaningful analysis and were excluded. For the women who had had more than one pregnancy, we chose the pregnancy nearest the cytology date. The final cohort consisted of 5,598 women.

Women had had term live births (≥37 weeks gestation, n=4942, 88.3%); all preterm birth (<37 weeks gestation, n=386, 6.9% [including those who had a late miscarriage, n=26]) and early miscarriage (<13 weeks gestation, n=270, 4.8% [obtained from SMR02, n=156, and SMR01, n=114]). On comparison with Scottish birth statistics ^23^, the age distribution of the cohort was much younger than that of the Scottish childbearing population. There were more pregnancy outcomes in women of <25 years (62.9%) than in the Scottish population ^23^ (26.5%) and a smaller proportion of mothers aged 25–34 years (28.8% vs. 53%) or ≥35 years (8.3% vs. 20.5%) in our cohort, respectively. The percentage of all women who had a preterm birth in our cohort (6.9%) was similar to that of the Scottish population ^23^, which ranged between 5.8 and 6.7% (between 1999 and 2015). The percentage of women having hospital admission for early miscarriage in our population was much lower (4.8%) than that in the Scottish birth data ^23^. Due to this low rate of miscarriage, the miscarriage data were deemed to be problematic due to uncertainty in case ascertainment and was thought to not be representative of the Scottish child-bearing population. Therefore, the results for early miscarriage are not presented.

The characteristics of the women according to the HPV viral/pathology parameters are shown in Table 5 for the whole cohort. The percentage of women with HR HPV-positive results in our cohort was 27.5%, which is higher than the HR-HPV prevalence (18%) seen previously in the Scottish screening population ^28^, probably due to the nature of ‘selective’ sampling in the Scottish HPV Archive. Similarly, the percentage of women with HPV-16/18 was higher than that in the routine prevalence in the same population (9.7% vs. 2.9%, respectively). For HPV-associated disease, some women were HR HPV-negative or of unknown genotype but showed evidence of HPV-associated disease. The proportion of women with low-grade disease was higher in our cohort than in the national screening data (16.0% vs. 7.5%, respectively) and our cohort had a higher percentage of women with high-grade cervical disease (2.9% vs. 1%, respectively).

**Table 5.** Maternal characteristics and HPV viral/pathology parameters in the cohort of women. Table shows the numbers and percentages of maternal characteristics and HPV viral/pathology parameters in the whole cohort of women, compared by Chi Square or Fisher’s Exact test. P values from results showing significant differences are highlighted in bold.

Maternal Characteristic ^†^	HPV viral/pathology parameter *											
	HR HPV (N=4061)			HR HPV Type (N=3519)				HPV-associated cervical disease (N=3884)				
	Negative, n (%)	Positive, n (%)	P value	Negative, n (%)	HPV16/18, n (%)	non-16 /non-18 HR HPV, n (%)	P value	HPV negative, n (%)	HR positive no disease, n (%)	Low-grade abnormalities, n (%)	High-grade disease, n (%)	P value
**Age (years)** ^‡^												
<20 years	666 (16.4)	347 (8.5)	**0.024**	666 (18.9)	122 (3.5)	111 (3.2)	0.211	623 (16.0)	95 (2.4)	208 (5.4)	23 (0.6)	**<0.001**
>20 years	1883 (46.4)	1165 (28.7)		1883 (53.5)	420 (11.9)	317 (9.0)		1751 (45.1)	360 (9.3)	685 (17.6)	139 (3.6)	
**Ethnicity** ^§^												
White	2492 (61.4)	1494 (36.8)	**0.017**	2492 (70.8)	535 (15.2)	425 (12.1)	0.052	2319 (59.7)	448 (11.5)	884 (22.8)	159 (4.1)	0.097
Other	57 (1.4)	18 (0.4)		57 (1.6)	<10 (<0.3)	<10 (<0.3)		55 (1.4)	<10 (<0.3)	<10 (<0.3)	<10 (<0.3)	
**Parity**												****
Non-parous	1752 (43.1)	1018 (25.1)	0.353	1752 (49.8)	390 (11.1)	310 (8.8)	0.139	1652 (42.5)	311 (8.0)	593 (15.3)	84 (2.2)	**<0.001**
Parous	797 (19.6)	494 (12.2)		797 (22.6)	152 (4.3)	118 (3.3)		722 (18.6)	144 (3.7)	300 (7.7)	78 (2.0)	
**SIMD** ^||^												
Most deprived	1248 (30.7)	818 (20.1)	**0.002**	1248 (35.5)	313 (8.9)	238 (6.8)	**<0.001**	1161 (29.9)	226 (5.8)	464 (11.9)	88 (2.3)	0.292
Least deprived	1301 (32.0)	694 (17.1)		1301 (36.9)	229 (6.5)	190 (5.4)		1213 (31.2)	229 (5.9)	429 (11.0)	74 (1.9)	
**BMI ^¶^ (kg/m ^2^)**												
<19.9	327 (8.1)	209 (5.1)		327 (9.3)	75 (2.1)	70 (2.0)		308 (7.9)	53 (1.4)	127 (3.3)	13 (0.3)	
20.0–24.9	969 (23.9)	614 (15.1)	0.122	969 (27.5)	215 (6.1)	176 (5.0)	0.132	897 (23.1)	182 (4.7)	372 (9.6)	78 (2.0)	**0.027**
25.0–29.9	741 (18.2)	392 (9.7)		741 (21.1)	138 (3.9)	106 (3.0)		686 (17.7)	118 (3.0)	242 (6.2)	39 (1.0)	
≥30 (obese)	512 (12.6)	297 (7.3)		512 (14.5)	114 (3.2)	76 (2.2)		484 (12.5)	102 (2.6)	152 (3.9)	31 (0.8)	
**Current Smoking**												
No	1854 (45.6)	1059 (26.1)	0.065	1854 (52.7)	359 (10.2)	302 (8.6)	**0.009**	1733 (44.6)	324 (8.3)	630 (16.2)	118 (3.0)	0.527
Yes	695 (17.1)	453 (11.2)		695 (19.7)	183 (5.2)	126 (3.6)		641 (16.5)	131 (3.4)	263 (6.8)	44 (1.1)	
**Diabetes** ^#^^§^												
No	2513 (61.9)	1489 (36.7)	0.787	2513 (71.4)	537 (15.3)	421 (12.0)	0.587	2340 (60.2)	447 (11.5)	879 (22.6)	157 (4.0)	0.423
Any	36 (0.9)	23 (0.5)		36 (1.0)	<10 (<0.3)	<10 (<0.3)		34 (0.9)	<10 (<0.3)	14 (0.4)	<10 (<0.3)	
**Hypertension** **												
No	2342 (57.7)	1409 (34.7)	0.142	2342 (66.5)	511 (14.5)	391 (11.1)	0.132	2175 (56.0)	422 (10.9)	829 (21.3)	152 (3.9)	0.503
Yes	207 (5.1)	103 (2.5)		207 (5.9)	31 (0.9)	37 (1.1)		199 (5.1)	33 (0.9)	64 (1.6)	10 (0.3)	
**HPV vaccination** **status** ^§^^††^												
Unvaccinated	1277 (36.1)	561 (15.8)		1277 (38.7)	353 (10.7)	208 (6.3)		1194 (35.9)	174 (5.2)	265 (8.0)	61 (1.8)	
Vaccinated	302 (8.5)	193 (5.5)		302 (9.1)	76 (2.3)	117 (3.5)		287 (8.6)	62 (1.9)	60 (1.8)	<10 (<0.3)	
Too old	762 (21.5)	446 (12.6)	**<0.001**	762 (23.1)	110 (3.3)	99 (3.0)	**<0.001**	718 (21.6)	166 (5.0)	239 (7.2)	98 (2.9)	**<0.001**

* The HPV viral/pathology parameters are not independent, so each woman has information for each parameter. Criteria for classifications for all HPV parameters are given in methods and Table 1. ^†^ Maternal characteristics used imputed data. ^‡^ The age cut off of 20 years was chosen because all of those who were eligible for vaccination were under 25 years of age. ^§^ Some groups contained numbers of women of <10 so were not released from the Safe Haven. ^||^ Scottish Index of Material Deprivation (SIMD) quintiles were combined as most deprived (quintiles 1&2) vs. least deprived (quintiles 3–5). ^¶^ Body mass index (BMI, kgm ^-2^) was calculated as weight (kg) divided by the square of the patient’s height (m) ^#^ Diabetes includes gestational diabetes and pre-existing diabetes ( Table 1). ** Hypertension did not distinguish between pre-existing or gestational hypertension within SMR02 ( Table 1). ^††^ Due to missing data, numbers for the HPV vaccination data do not add up to the totals given.

### Analysis of the cohort

The numbers and percentages for the maternal factors were compared by pregnancy outcomes (term live births vs. all preterm birth, Table 4). There were no significant differences in maternal age, ethnicity, parity, SIMD (most vs. least deprived), BMI or hypertension between women who had term live births compared with those who had preterm births. A higher proportion of all women who had a preterm birth were current smokers when compared with those who had term live births (P<0.001). Similarly, a higher proportion of all women who had a preterm birth also had diabetes than those who had term live birth (P=0.063, Table 4).

The numbers and percentages of women in each analysis and the unadjusted and adjusted OR from binary logistic regression are shown for each HPV viral/pathology parameter and all women who had a preterm birth, for all women ( Table 6) and vaccine-eligible women ( Table 7); similarly results for spontaneous preterm birth are shown in Table 8 and Table 9.

**Table 6.** Any preterm birth compared with term live-birth in all women, according to HPV viral/pathological parameters. Table shows the pregnancy outcomes: term live births (>37 weeks gestation) and any preterm birth (<37 weeks gestation) compared by each HPV parameter for all women in the cohort. The numbers and percentages in each group are shown. The odds ratios (OR) and P values from unadjusted and adjusted binary logistic regression models are shown. Significant associations are highlighted in bold.

HPV Parameter	Term live birth, n (%) *	Any preterm birth, n (%) *	Results from logistic regression models	
			Unadjusted OR (95% CI) p value	Adjusted † OR (95% CI) p value
**HR HPV**				
Negative	2281 (93.3)	165 (6.7)	Reference	Reference
Positive	1302 (91.4)	122 (8.6)	**1.295 (1.015–1.653) 0.037**	1.260 (0.985–1.612) 0.066
**HPV16/18 type**				
HR Negative	2281 (93.3)	165 (6.7)	Reference	Reference
HPV16/18	471 (91.5)	44 (8.5)	1.291 (0.913–1.827) 0.149	1.255 (0.883–1.784) 0.205
Non16/non18 HR HPV	369 (91.8)	33 (8.2)	1.236 (0.837–1.825) 0.286	1.174 (0.792–1.740) 0.424
**HPV-associated** **cervical disease** ‡				
HR negative	2123 (93.3)	153 (6.7)	Reference	Reference
HR HPV+ve no disease	387 (92.4)	32 (7.6)	1.147 (0.772–1.702) 0.496	1.110 (0.745–1.655) 0.608
Low-grade abnormalities	773 (92.7)	61 (7.3)	1.095 (0.805–1.490) 0.564	1.061 (0.777–1.448) 0.710
High-grade disease	140 (88.1)	19 (11.9)	**1.883 (1.135–3.125) 0.014**	**1.843 (1.101–3.083) 0.020**

* Due to missing HPV data, numbers and percentages relate to the numbers that were present in each analysis and do not add up to the total numbers of women in each pregnancy outcome. ^†^ Adjusted binary logistic regression models used the imputed dataset and are pooled results from 5 imputations. Models were adjusted for ethnicity, SIMD (most deprived vs. least deprived), maternal age, parity, smoker in current pregnancy, diabetes, hypertensive disorder and maternal BMI. ^‡^ Criteria for HPV-associated cervical disease are shown in the methods and Table 1. Cofactors that had a significant association with preterm birth from these models were: **HR HPV**: Current Smoking OR=1.429 (1.096–1.863) P=0.008; Diabetes OR=2.745 (1.280–5.884) P=0.010; Hypertension OR=1.552 (1.045–2.305) P=0.030. **HR HPV16/18 type**: Current Smoking OR=1.367 (1.018–1.836) P=0.038; Diabetes OR=3.243 (1.414–7.441) P=0.006; Hypertension OR=1.609 (1.051–2.462) P=0.029. **HPV-associated cervical disease**: Current Smoking OR=1.573 (1.193–2.073) P=0.001; Diabetes OR=2.641 (1.271–5.489) P=0.009; Maternal age ≤20 years OR=0.686 (0.495–0.950) P=0.023.

**Table 7.** Any preterm birth compared with term live-births in vaccine-eligible women, according to HPV viral/pathological parameters. Table shows the women with each pregnancy outcome: term live births (>37 weeks gestation) and any preterm birth (<37 weeks gestation) compared by each HPV parameter for vaccine-eligible women in the cohort. The numbers and percentages in each group are shown. The odds ratios (OR) and P values from unadjusted and adjusted binary logistic regression models are shown. Significant associations are highlighted in bold.

HPV Parameter	Term live birth *, n (%)	Any preterm birth *, n (%)	Results from logistic regression	
			Unadjusted OR (95% CI) p value	Adjusted ^†^ OR (95% CI) p value
**HR HPV**				
HR HPV negative	1583 (93.2)	115 (6.8)	Reference	Reference
HR HPV Positive	905 (91.2)	87 (8.8)	1.323 (0.990–1.769) 0.059	1.282 (0.956–1.719) 0.098
*With adjustment for* *Vaccination status*				
*HR HPV negative*	1583 (93.2)	115 (6.8)	Reference	Reference
*HR HPV Positive*	905 (91.2)	87 (8.8)	1.310 (0.943–1.820) 0.108	1.255 (0.899–1.752) 0.182
*Unvaccinated*	2026 (92.6)	163 (7.4)	Reference	Reference
*Vaccinated*	510 (93.4)	36 (6.6)	0.884 (0.591–1.320) 0.545	0.925 (0.613–1.395) 0.710
**HPV16/18 type**				
HR HPV negative	1583 (93.2)	115 (6.8)	Reference	Reference
HR HP16/18	371 (91.2)	36 (8.8)	1.336 (0.903–1.975) 0.147	1.312 (0.883–1.948) 0.179
Non-16/non18 HR HPV	280 (91.2)	27 (8.8)	1.327 (0.857–2.057) 0.205	1.247 (0.801–1.943) 0.328
*With adjustment for* *Vaccination status*				
*HR negative*	1583 (93.2)	115 (6.8)	Reference	Reference
*HPV 16/18*	371 (91.2)	36 (8.8)	1.282 (0.859–1.912) 0.224	1.252 (0.836–1.875) 0.276
*Non16/non18 HR HPV*	280 (91.2)	27 (8.8)	1.349 (0.863–2.109) 0.188	1.260 (0.801–1.982) 0.318
*Unvaccinated*	2026 (92.6)	163 (7.4)	Reference	Reference
*Vaccinated*	510 (93.4)	36 (6.6)	0.879 (0.587–1.317) 0.533	0.925 (0.611–1.399) 0.710
**HPV-associated Cervical** **Disease ^‡^^§^**				
HR negative	1464 (93.2)	107 (6.8)	Reference	Reference
HR HPV+ve no disease	236 (91.5)	22 (8.5)	1.275 (0.790–2.059) 0.319	1.251 (0.772–2.029) 0.363
Low-grade abnormalities	561 (93.0)	42 (7.0)	1.024 (0.708–1.483) 0.899	0.980 (0.674–1.426) 0.918
High-grade disease	-- (89.0)	-- (11.0)	1.710 (0.761–3.844) 0.194	1.577 (0.693–3.586) 0.277
*With adjustment for* *Vaccination status*				
*HR negative*	1464 (93.2)	107 (6.8)	Reference	Reference
*HR HPV+ve no disease*	236 (91.5)	22 (8.5)	1.275 (0.754–2.158) 0.365	1.223 (0.718–2.084) 0.458
*Low-grade abnormalities*	561 (93.0)	42 (7.0)	0.989 (0.600–1.629) 0.965	0.935 (0.564–1.549) 0.793
*High-grade disease*	-- (89.0)	-- (11.0)	1.673 (0.741–3.799) 0.216	1.506 (0.655–3.463) 0.335
*Unvaccinated*	2026 (92.6)	163 (7.4)	Reference	Reference
*Vaccinated*	510 (93.4)	36 (6.6)	0.823 (0.518–1.308) 0.410	0.863 (0.538–1.386) 0.543

* Due to missing HPV data, numbers and percentages relate to the numbers that were present in each analysis and do not add up to the total numbers in each pregnancy outcome. ^**†**^ Adjusted binary logistic regression models used the imputed dataset and are pooled results from 5 imputations. Models were adjusted for ethnicity, SIMD (most deprived vs. least deprived), maternal age, parity, smoker in current pregnancy, diabetes, hypertensive disorder and maternal BMI. ^**‡**^ Criteria for HPV-associated cervical disease are shown in the methods and Table 1. ^**§**^ Where there were groups containing <10 women, the numbers were not released from the Safe Haven and only the percentages are given. Cofactors that had a significant association with preterm birth from these models were: **HR HPV**: without adjustment for vaccination Diabetes OR=3.552 (1.495–8.442), P=0.004; with adjustment for vaccination Diabetes OR=4.479 (1.834–10.94) P=0.001. **HR HPV16/18 type**: without adjustment for vaccination Diabetes OR=4.158 (1.714–10.09) P=0.002; with adjustment vaccination Diabetes OR=4.478 (1.832–10.95) P=0.001. **HPV-associated cervical disease**: without adjustment for vaccination Diabetes OR=3.529 (1.479–8.422) P=0.004; with adjustment for vaccination Diabetes OR=4.992 (1.999–12.46) P=0.001, Maternal age ≤20 years OR=0.669 (0.448–0.998) P=0.049.

**Table 8.** Spontaneous preterm birth compared with term live-birth in all women, according to HPV viral/pathological parameters. Table shows the women who had pregnancy outcomes: term live births (>37 weeks gestation) and spontaneous preterm birth (<37 weeks gestation) compared by each HPV parameter for all women. The numbers and percentages in each group are shown. The odds ratios (OR) and P values from unadjusted and adjusted binary logistic regression models are shown. Significant associations are highlighted in bold.

HPV Parameter	Term live birth, n (%) *	Any preterm birth, n (%) *	Results from logistic regression models	
			Unadjusted OR (95% CI) p value	Adjusted † OR (95% CI) p value
**HR HPV**				
Negative	2281 (94.2)	140 (5.8)	Reference	Reference
Positive	1302 (93.0)	98 (7.0)	1.226 (0.939–1.602) 0.134	1.189 (0.908–1.556) 0.209
**HPV16/18 type**				
HR Negative	2281 (94.2)	140 (5.8)	Reference	Reference
HPV16/18	471 (93.1)	35 (6.9)	1.291 (0.825–1.777) 0.328	1.169 (0.794–1.723) 0.429
Non16/non18 HR HPV	369 (92.7)	29 (7.3)	1.280 (0.846–1.939) 0.243	1.220 (0.803–1.854) 0.352
**HPV-associated** **cervical disease ‡**				
HR negative	2123 (94.2)	130 (5.8)	Reference	Reference
HR HPV+ve no disease	387 (93.3)	28 (6.7)	1.182 (0.774–1.803) 0.439	1.141 (0.746–1.746) 0.544
Low-grade abnormalities	773 (93.9)	50 (6.1)	1.056 (0.755–1.479) 0.750	1.015 (0.723–1.426) 0.930
High-grade disease	140 (89.7)	16 (10.3)	**1.866 (1.080–3.224) 0.025**	**1.791 (1.028–3.121) 0.040**

* Due to missing HPV data, numbers and percentages relate to the numbers that were present in each analysis and do not add up to the total numbers in each pregnancy outcome. ^†^ Adjusted binary logistic regression models used the imputed dataset and are pooled results from 5 imputations. Models were adjusted for ethnicity, SIMD (most deprived vs. least deprived), maternal age, parity, smoker in current pregnancy, diabetes, hypertensive disorder and maternal BMI. Any cofactors that had a significant association with preterm birth from these models are shown. ^**‡**^ Criteria for HPV-associated cervical disease are shown in the methods. Cofactors that had a significant association with preterm birth from these models were: **HR HPV**: Current smoking OR=1.448 (1.079–1.942), P=0.014 **HR HPV16/18 type**: Current Smoking OR=1.445 (1.050–1.990), P=0.024 **HPV-associated cervical disease**: Current Smoking OR=1.599 (1.182–2.162) P=0.002; Diabetes OR=2.499 (1.088–5.738) P=0.031; Maternal Age ≤20 years OR=0.662 (0.464–0.945) P=0.023.

**Table 9.** Spontaneous preterm birth compared with term live-births in vaccine-eligible women, according to HPV viral/pathological parameters. Table shows the pregnancy outcomes term live births (>37 weeks gestation) and spontaneous preterm birth (<37 weeks gestation) compared by each HPV parameter for vaccine-eligible women. The numbers and percentages in each group are shown. The odds ratios (OR) and P values from unadjusted and adjusted binary logistic regression models are shown. Significant associations are highlighted in bold.

HPV Parameter	Term live birth *, n (%)	Any preterm birth *, n (%)	Results from logistic regression	
			Unadjusted OR (95% CI) p value	Adjusted † OR (95% CI) p value
**HR HPV**				
HR HPV negative	1583 (94.3)	95 (5.7)	Reference	Reference
HR HPV Positive	905 (93.0)	68 (7.0)	1.252 (0.907–1.727) 0.171	1.210 (0.874–1.674) 0.251
*With adjustment for* *Vaccination status*				
*HR HPV negative*	1583 (94.3)	95 (5.7)	Reference	Reference
*HR HPV Positive*	905 (93.0)	68 (7.0)	1.300 (0.908–1.862) 0.151	1.256 (0.874–1.806) 0.218
*Unvaccinated*	2026 (93.5)	140 (6.5)	Reference	Reference
*Vaccinated*	510 (95.1)	26 (4.9)	0.740 (0.467–1.174) 0.202	0.756 (0.472–1.211) 0.244
**HPV16/18 type**				
HR HPV negative	1583 (94.3)	95 (5.7)	Reference	Reference
HR HP16/18	371 (93.0)	28 (7.0)	1.258 (0.813–1.946) 0.303	1.230 (0.792–1.910) 0.358
Non-16/non18 HR HPV	280 (92.1)	24 (7.9)	1.428 (0.897–2.275) 0.133	1.348 (0.843–2.155) 0.213
*With adjustment for* *Vaccination status*				
*HR negative*	1583 (94.3)	95 (5.7)	Reference	Reference
*HPV 16/18*	371 (93.0)	28 (7.0)	1.179 (0.754–1.843) 0.470	1.156 (0.737–1.814) 0.528
*Non16/non18 HR HPV*	280 (92.1)	24 (7.9)	1.474 (0.917–2.370) 0.109	1.396 (0.864–2.256) 0.173
*Unvaccinated*	2026 (93.5)	140 (6.5)	Reference	Reference
*Vaccinated*	510 (95.1)	26 (4.9)	0.725 (0.455–1.154) 0.175	0.742 (0.461–1.193) 0.218
**HPV-associated Cervical** **Disease** ‡ §				
HR negative	1464 (94.3)	88 (5.7)	Reference	Reference
HR HPV+ve no disease	236 (92.5)	19 (7.5)	1.339 (0.801–2.241) 0.266	1.319 (0.785–2.216) 0.295
Low-grade abnormalities	561 (94.3)	34 (5.7)	1.008 (0.671–1.516) 0.968	0.959 (0.635–1.449) 0.843
High-grade disease	-- (90.3)	-- (9.7)	1.782 (0.748–4.250) 0.192	1.625 (0.673–3.922) 0.280
*With adjustment for* *Vaccination status*				
*HR negative*	1464 (94.3)	88 (5.7)	Reference	Reference
*HR HPV+ve no disease*	236 (92.5)	19 (7.5)	1.372 (0.785–2.397) 0.267	1.326 (0.755–2.330) 0.327
*Low-grade abnormalities*	561 (94.3)	34 (5.7)	1.126 (0.671–1.888) 0.654	1.066 (0.632–1.798) 0.812
*High-grade disease*	-- (90.3)	-- (9.7)	1.687 (1.704–4.041) 0.241	1.518 (0.622–3.701) 0.359
*Unvaccinated*	2026 (93.5)	140 (6.5)	Reference	Reference
*Vaccinated*	510 (95.1)	26 (4.9)	0.689 (0.407–1.168) 0.167	0.719 (0.420–1.231) 0.229

* Due to missing HPV data, numbers and percentages relate to the numbers that were present in each analysis and do not add up to the total numbers in each pregnancy outcome. † Adjusted binary logistic regression models used the imputed dataset and are pooled results from 5 imputations. Models were adjusted for ethnicity, SIMD (most deprived vs. least deprived), maternal age, parity, smoker in current pregnancy, diabetes, hypertensive disorder and maternal BMI. Any cofactors that had a significant association with preterm birth from these models are shown. ‡ Criteria for HPV-associated cervical disease are shown in the methods. § Where there were groups containing <10 women, the numbers were not released from the Safe Haven and only the percentages are given. Cofactors that had a significant association with preterm birth from these models were: **HR HPV**: without adjustment for vaccination status there were no significant cofactors; with adjustment for vaccination status Diabetes OR=3.190 (1.054–9.652) P=0.040 **HR HPV16/18 type**: without adjustment for vaccination status there were no significant cofactors; with adjustment for vaccination status Diabetes OR=3.165 (1.045–9.586), P=0.042. **HPV-associated cervical disease**: without adjustment for vaccination Diabetes OR=3.113 (1.146–8.456) P=0.026; with adjustment for vaccination status Diabetes OR=4.270 (1.513–12.05) P=0.006

***HR HPV.*** Binary logistic regression showed that women who carried any HR HPV had increased odds of having a preterm birth in unadjusted models, but the association was lost in adjusted models ( Table 6). In vaccine-eligible women there was no association between HR HPV and all preterm birth and no effect of vaccine ( Table 7). Similarly, for spontaneous preterm birth, there was no association between HR HPV and spontaneous preterm birth in the whole cohort ( Table 8), nor in vaccine-eligible women and no effect of vaccine ( Table 9). Current smoking and/or diabetes were independently associated with increased odds of all preterm birth and/or spontaneous preterm birth. Hypertension was an independent predictor of all preterm birth in all women but not vaccine-eligible women.

***HR HPV 16/18 types.*** Only 970 women had known specific HR HPV types. There were no associations between carriage of HPV16/18 types or non16/non18 HPV types and all preterm birth ( Table 6 and Table 7) or spontaneous preterm birth ( Table 8 and Table 9) in the whole cohort nor in vaccine-eligible women, and vaccination had no effect. Current smoking and/or diabetes were independently associated with increased odds of any and/or spontaneous preterm birth. Hypertension was an independent predictor of any preterm birth in all women but not vaccine-eligible women.

***HPV-associated cervical disease.*** In the whole cohort of women, high-grade cervical disease was significantly associated with ~80% increased odds of any and spontaneous preterm birth ( Table 6 and Table 8, respectively) in unadjusted and adjusted models of all women. In the vaccine-eligible women there were no associations between any level of HPV-associated disease and no effect of vaccination in any preterm birth or spontaneous preterm birth ( Table 7 and Table 9, respectively). Current smoking and diabetes were also associated with an increased odds of any or spontaneous preterm birth in all women. Younger maternal age showed a decreased association with HPV-associated cervical disease in the models for both any and spontaneous preterm birth.

## Discussion

This study found associations between different HPV viral/pathology parameters and adverse pregnancy outcomes. Specifically, high-grade HPV-associated cervical disease was associated with preterm birth and spontaneous preterm birth in the whole cohort women, but not in the vaccine-eligible subgroup of younger women. No associations were seen between HR HPV-positive (with no disease) or low-grade HPV-associated cervical disease and all or spontaneous preterm birth.

Associations between high-grade cervical disease and all preterm birth or spontaneous preterm birth have been suggested in previous studies ^13, 14^. In this study, such associations were not seen in the younger, vaccine-eligible subgroup of women, suggesting that the older women were infected for longer and had time to develop high-grade cervical disease ^31^. Previous studies showing associations between HPV-associated cervical disease and preterm birth have been ambiguous regarding whether these were due to HPV-infection itself, development of associated CIN lesions or the excision of these lesions ^32, 33^. Low-grade cervical lesions are more likely to be associated with productive HPV-infections, where the viral life-cycle is completed, compared with infections associated with severe disease where the viral life-cycle is poorly supported ^34^. Productive infections are also associated with a peak/burst of viral load over a narrower time-frame than infections associated with high-grade disease ^35^. Therefore, in the absence of any associations with HR HPV-infection alone or low-grade cervical disease, and given that over 90% of those who had high-grade disease would have been treated ^4^, we suggest that the association with high-grade cervical disease, in our cohort, is likely to be due to the treatment of HPV-associated cervical disease rather than a direct effect of HPV-infection itself. The clinical implication is that women who have undergone treatment for HPV-associated cervical disease should be closely monitored in subsequent pregnancies for any signs of preterm birth.

Although only HPV-associated high-grade cervical disease was associated with all preterm birth and spontaneous preterm birth, current smoking was consistently associated with spontaneous preterm birth. Smoking is a previously recognised risk factor for spontaneous preterm birth ^36^. Diabetes and hypertension were also independently associated with all preterm birth, but this was not surprising as these are likely to be the indicative reasons for early delivery of the baby. A recent study suggested that HPV infection in the placenta was associated with a number of pregnancy complications ^37^, including gestational diabetes. However, our data did not find an increased association between HPV viral/pathology data and diabetes and so do not support such an association.

There are strengths and weaknesses for this study. The strengths of this study are that this was a data linkage study starting with women for whom HPV-infection status was known. The use of the Scottish HPV Archive meant that the HPV viral/pathology parameters for these women had been robustly characterised and the HPV information was not available from any other source. The use of routinely collected data should also reduce bias in the recording of clinical information. Primary HPV screening in Scotland will soon include results from HPV testing. Such information may facilitate a future population-wide analysis of the effects of HPV-infection and pregnancy outcomes in the Scottish population.

A weakness of the study is that we did not have details of any treatments for cervical disease, so we have had to rely on an assumption that women with high-grade disease received excisional treatment as per national guidelines. The age-distribution of the women did not reflect that of the Scottish childbearing population ^23^, and therefore, it is hard to determine how representative these results are of the wider population. The study cohort was drawn from the general population by the fact that they underwent cervical screening, but were considerably younger at pregnancy outcome and had higher proportions of women with HPV-infection and HPV-associated cervical disease, due to the selective nature of the samples within the Scottish HPV Archive. The preterm birth rates were similar to that in the Scottish population. The early miscarriage data were obtained from maternity/hospital admission records, but would not include miscarriages managed at home, through outpatient and emergency departments or by General Practitioners. The risk of miscarriage increases with age, particularly after the age of 40 years, although a slightly increased risk of miscarriage exists in women aged under 20 years ^38^. The miscarriage rate in our population was much lower than the that reported for miscarriages managed in hospitals ^23^. While this lower rate may particularly reflect the youth of our cohort, the differences are hard to explain, and so these data were not presented.

In summary, our data linkage study has found no evidence that HPV infection per se, or low-grade HPV-associated cervical disease was associated with preterm birth. High-grade disease, the majority of which is treated with excisional treatments, was associated with spontaneous preterm birth. This is consistent with previous evidence linking excisional cervical treatment with increased risk of spontaneous preterm birth ^13^.

## Data availability

### Underlying data

The linked dataset was analysed within the NHS National Safe Haven, provided by NHS Research Scotland. The Safe Haven is a remote server through which the researcher accesses the health data and services to enable research while protecting the confidentiality of the data. Data remains under the control of the NHS and complies with legislative and NHS policies. The linked dataset is archived within the Safe Haven and is available by application to NHS Scotland via the electronic Data Research and Innovation Service (eDRIS), within NHS National Services Scotland ( https://www.isdscotland.org/Products-and-Services/eDRIS/).

To apply for access to these data, please read the guide for researchers and then complete the Enquiry Form, describing your planned study and the data required, and email it to nss.edris@nhs.net.

### Reporting guidelines

Figshare: RECORD checklist for “HPV infection and pre-term birth: a data-linkage study using Scottish Health Data”. https://doi.org/10.6084/m9.figshare.7764755.v1 ^39^.
