# Supplementary material for: HPV infection and pre-term birth: a data-linkage study using Scottish Health Data
Source: Wellcome Open Res. 2019 Mar 8;4:48. [Version 1] doi: 10.12688/wellcomeopenres.15140.1 (PMC6436145; doi:10.12688/wellcomeopenres.15140.1)
Supplement: Peer review history

# Peer review: Reviewer response for version 1

**DOI:** 10.21956/wellcomeopenres.16519.r35034

**Published:** 2019-03-29

**Authors:** Fay Crawford

**Affiliations:** 1 Queen Margaret Hospital, NHS Fife, Dunfermline, UK

This data linkage study has analysed data for associations between HPV infections and/or HPV-associated cervical disease and pre-term birth. 

 The researchers included data from a Scottish population of 5,626 women who are not entirely representative of the Scottish childbearing population. However, this and other weaknesses of the study are candidly discussed.

 The finding that poor maternal outcomes are not associated with HPV infection but high-grade cervical disease converges with other studies on the subject but is interesting and important to report in this data registry population.  

 The association between smoking, diabetes and hypertension and poor pregnancy are known but it is useful to have these results added to the evidence base. 

 The paper is very well-written and clear.

I have read this submission. I believe that I have an appropriate level of expertise to confirm that it is of an acceptable scientific standard.

# Peer review: Reviewer response for version 1

**DOI:** 10.21956/wellcomeopenres.16519.r35031

**Published:** 2019-03-25

**Authors:** Ronnie Lamont

**Affiliations:** 1 Department of Surgery, University College London (UCL), London, UK

**Originality:**

 I am not aware of any similar registry based study.

**Clinical importance:**

 Preterm birth (PTB) is an important area of research because it is the major cause of neonatal mortality and morbidity worldwide, and infection is a major cause, particularly at low gestational ages.

**Scientific merit:**

 I am not qualified to comment on the use of registry data, the statistical methods in general or the use of imputation techniques in particular. Otherwise I have no problem with the conduct or methodology of the study.

**Comments**

1. Bearing in mind the paucity of research in his area, the authors should consider citing the review of adverse outcomes of pregnancy in association with HPV ( *Bonde U, et al. *The potential role of HPV vaccination in the prevention of infectious complications of pregnancy ^1^.
2. The authors should consider alluding to the safety or otherwise of HPV vaccination in pregnancy ( *Bonde U, et al. *Is HPV vaccination in pregnancy safe? ^2^). 
3. The study by Bruinsma (cited as Reference 14) had significant flaws and these were highlighted in the subsequent correspondence(Lamont RF, Sarhanis P. Precancerous changes in the cervix and risk of subsequent preterm birth ^3^). This should be mentioned.
4. Although well defined in the main text, the term “cervical disease” is nebulous in the context of the abstract. If the word count permits, it would be worth making this clearer in the abstract. 
5. I had to search for the definition of “vaccine eligible” and found this in the last part of Table 1. To appreciate the importance of the 1 ^st ^two paragraphs of the discussion, it would be helpful for this to be defined in the methods section.
6. I found Table 5 very confusing, particularly with respect to vaccination status compared to HPV viral/pathology parameters. The statistically significant results may need to be spelled out/expanded in the text rather than having to be gleaned from the Table.
7. I had to read the first two paragraphs of the discussion half a dozen times before I understood the message. This might be due to my ignorance and may become more clear once comments 5 and 6 above are addressed, but I think could be made clearer if expanded. There must be better ways of making terms such as “vaccine-eligible sub-group of younger women”, and “productive HPV-infections”, better understood.
8. In the study, the outcome of PTB was a categorical variable. Was it possible to stratify the gestational age of PTB in relation to HR-HPV and cervical disease? In other words, if PTB was considered as a continuous variable was there suffficieent data to demonstrate an association between HR-HPV, cervical disease and extreme, early or late PTB?
9. The authors record that current smoking was independently associated with an increased risk of PTB, but did not speculate on the link. One possibility is through temporate phage viruses being provoked to become lytic by cigarette smoke carcinogens in cervicovaginal secretions. Diet acquired phage viruses, may be induced to become lytic by factors related to sexual activity, and *Lactobacillus*phage viruses may be directly inoculated into the vagina from male or female partners. Benzo-(a)-pyrene-dio-epoxide (BPDE), the metabolite of a chemical carcinogen in cigarette smoke, can be found in the vaginal secretions of women who smoke. With increasing concentrations of BPDE, there is an increase in the frequency of phage released. Cigarette smoke chemicals promote phage induction in vaginal lactobacilli *, *and these phages lyse other vaginal *Lactobacillus *strains. Tobacco products can be found in the cervical secretions of women with CIN and in the semen of smokers. Bacterial vaginosis is more common in women who smoke and both BV and smoking are linked by common sequelae such as PTB. It is therefore possible that female smokers secrete, or their partners who smoke, may introduce, by sexual transmission, tobacco products like BPDE into the vagina, which puts them at greater risk of BV by inducing endogenous or sexually acquired *Lactobacillus *phages to become lytic and an increased the risk of PTB. 
10. The clear message to me was, that PTB in association with HPV and CIN, was more likely to be due to the treatment of HR-HPV with HGCIN, than the presence of HR-HPV and HGCIN *per se. *This did not become clear to me until the last sentence of the discussion. Could this be made clearer earlier in the manuscript.

I have read this submission. I believe that I have an appropriate level of expertise to confirm that it is of an acceptable scientific standard.

## References

[1] BondeUJoergensenJSMogensenOLamontRF: The potential role of HPV vaccination in the prevention of infectious complications of pregnancy. *Expert Rev Vaccines*.2014;13(11) : 10.1586/14760584.2014.944164 1307-16 10.1586/14760584.2014.944164 25084626

[2] BondeUJoergensenJLamontRMogensenO: Is HPV vaccination in pregnancy safe?. *Human Vaccines & Immunotherapeutics*.2016;12(8) : 10.1080/21645515.2016.1160178 1960-1964 10.1080/21645515.2016.1160178 PMC499472327172372

[3] BruinsmaFLumleyJTanJQuinnM: Precancerous changes in the cervix and risk of subsequent preterm birth. *BJOG*.2007;114(1) : 10.1111/j.1471-0528.2006.01107.x 70-80 10.1111/j.1471-0528.2006.01107.x 17083653

# Peer review: Reviewer response for version 1

**DOI:** 10.21956/wellcomeopenres.16519.r35032

**Published:** 2019-03-11

**Authors:** Pierre L. Martin-Hirsch

**Affiliations:** 1 Department of Obstetrics and Gynaecology, Lancashire Teaching Hospitals NHS Foundation Trust, Preston, UK

This study is an important and timely contribution to the evidence base around the influence of oncogenic HPV subtypes and pre-term labour. It is excellently written. I am not an expert in statistics but the methodology looks fine. Previous reports by Bruinsma cited by the author have suggested that the presence of dysplasia of all types potentially increases the risk of preterm labour whilst there is clear evidence that excisions treatment significantly increases risk particularly if excisions are of excessive volume/depth.

 This linkage study from robust databases within the Scottish health care system relating HPV status and pregnancy outcomes is very timely as it clearly demonstrates that having HPV infection alone without treatment does not influence pregnancy outcomes .

 There is growing evidence that observational management for low and intermediate grade cervical precursors leads to high rate of spontaneous regression and therefore excisional treatment can be avoided. Women can have the re-assurance that their risk of adverse pregnancy outcomes is not increased by HPV transient disease.  Furthermore, with the advent of primary HPV screening, when significant proportion of women are going to found to be High risk HPV positive but disease free, can also be re-assured by this data that this will not increase the risk of pre-term delivery etc.

 I think this study is an important contribution and should be shared with individuals designing information resources for women regarding HPV testing etc.

I have read this submission. I believe that I have an appropriate level of expertise to confirm that it is of an acceptable scientific standard.
